# The role of GATA family transcriptional factors in haematological malignancies: A review: Retraction

**DOI:** 10.1097/MD.0000000000038232

**Published:** 2024-05-03

The article “The role of GATA family transcriptional factors in haematological malignancies: A review”,^[1]^ which appeared in Volume 103, Issue 12 of *Medicine®*, is being retracted due to concerns over some text used without proper attribution from “GATA family transcriptional factors: emerging suspects in hematologic disorders”^[2]^ and “GATA Transcription Factors and Cancer”^[3]^ as well as the unauthorized use of Figure 1, which originally appeared in “The GATA factor revolution in hematology.”^[4]^
